# Supplementary material for: Optomechanics with a hybrid carbon nanotube resonator
Source: Nat Commun. 2018 Feb 14;9:662. doi: 10.1038/s41467-018-03097-z (PMC5813052; doi:10.1038/s41467-018-03097-z)
Supplement: Supplementary file 1 — Supplementary Information [file 41467_2018_3097_MOESM1_ESM.pdf]

Optomechanics with a Hybrid Carbon Nanotube Resonator

Tavernarakis et al.

Supplementary Information

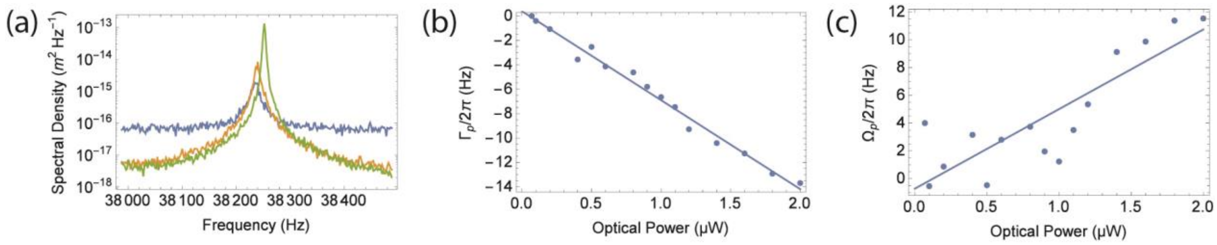

Supplementary Figure 1 : **Dynamical backaction amplification.** (a) Calibrated optomechanical fluctuation spectra acquired with the hybrid CNT resonator being set in the middle of the right "detection lobe" ($x_0 = w_0/2$). The three, increasing peaks are acquired for an input power of $70\,nW$, $1.1\,\mu W$ and $3\,\mu W$, respectively. (b) Evolution of the photothermal damping rate Γ_p as a function of the input optical power. Each dot is obtained by fitting the related optomechanical spectrum. A decreasing linear trend is observed (solid line). (c) Evolution of the photothermal frequency shift $\delta\Omega_p$ as a function of the input optical power. Each dot is obtained by fitting the related optomechanical spectrum. An increasing linear trend is observed (solid line).

Supplementary Note 1

Thermal properties and morphology

Our analysis of the dissipative component of the dynamical backaction enables to determine an effective thermal conductivity $\kappa_{\mathrm{eff}} \simeq 12.7\ \mathrm{W\,m^{-1}K^{-1}}$, two orders of magnitude below other reports. This reduced value can be explained by the presence of an amorphous carbon shell around the CNT resonator. The equivalent conductance of such system is obtained as the sum of its components, $G_{\mathrm{eq}} = G_0 + G_{\mathrm{shell}}$, with $G_{eq} = \frac{S\kappa_{\mathrm{eff}}}{L}$ (L the length, S the cross section area and κ_{eff} the effective conductivity of the total system), $G_0 = \frac{S_0\kappa_0}{L}$ the conductance of the CNT resonator (S_0 its cross section area and $\kappa_0 \simeq 1000\ \mathrm{W\,m^{-1}K^{-1}}$ its thermal conductivity) and $G_{\mathrm{shell}} = \frac{S_1\kappa_1}{L}$ the conductance of the amorphous carbon shell (S_1 its cross section area and $\kappa_1 \simeq 0.1\ \mathrm{W\,m^{-1}K^{-1}}$ its thermal conductivity). The effective conductivity can therefore be expressed as $\kappa_{\mathrm{eff}} = \frac{S_0}{S}\kappa_0 + \frac{S_1}{S}\kappa_1$. Noting that $S = S_0 + S_1$, we finally obtain $S = \frac{\kappa_0-\kappa_1}{\kappa_{\mathrm{eff}}-\kappa_1}S_0 \simeq \frac{\kappa_0}{\kappa_{\mathrm{eff}}}S_0$, and $r_{\mathrm{shell}} = \sqrt{\frac{S-S_0}{\pi}} \simeq 3.9\ \mathrm{nm}$ (where we have assumed a diameter for the pristine tube of $1\ \mathrm{nm}$).

Supplementary Note 2

Platinum nanoparticle mass and geometry

Due to the very large thermal fluctuations of the hybrid carbon nanotube resonator, it is difficult to infer the mass and geometry of the platinum nanoparticle from conventional imaging techniques. To estimate the mass, we therefore rely on the optomechanical calibration reported in the main manuscript. From the calibrated displacements, we obtain an effective mass $m_{\mathrm{eff}} = 0.79 \times 10^{-18}\mathrm{kg}$, hich is the sum of 3 contributions, $m_{\mathrm{eff}} = m_{\mathrm{CNT}} + m_{\mathrm{Pt}} + m_{\mathrm{shell}}$, with $m_{\mathrm{CNT}} = \frac{\pi[r_{\mathrm{CNT}}^2-(r_{\mathrm{CNT}}-G)^2]L\rho_{\mathrm{C}}^2}{4} \simeq 2 \times 10^{-20}\mathrm{kg}$ (with $r_{\mathrm{CNT}} \simeq 0.5\ \mathrm{nm}$ the radius of the carbon nanotube resonator, $L \simeq 5\ \mu\mathrm{m}$ its length, $G \simeq 0.34\ \mathrm{nm}$ the wall thickness and $\rho_{\mathrm{C}} \simeq 2000\ \mathrm{kg\,m^{-3}}$ the mass density of Carbon), m_{Pt} the mass of the Platinum nanoparticle and

$m_{\mathrm{shell}} \simeq \frac{[(r_{\mathrm{shell}}+r_{\mathrm{CNT}})^2-r_{\mathrm{CNT}}^2]L\rho_{\mathrm{C}}}{4} \simeq 1.5 \times 10^{-19}\mathrm{kg}$. This represents 80% (resp. 52%) of the motional (resp. physical) mass of the device. Further assuming a cylindrical geometry, the thickness $t_{\mathrm{Pt}} \simeq \sqrt{(r_{\mathrm{shell}} + r_{\mathrm{CNT}})^2 + \frac{m_{\mathrm{Pt}}}{\pi L_{\mathrm{Pt}}\rho_{\mathrm{Pt}}}} - (r_{\mathrm{shell}} + r_{\mathrm{CNT}}) \simeq 1.5\ \mathrm{nm}$, with $L_{\mathrm{Pt}} \simeq 575\ \mathrm{nm}$ the length of the nanoparticle (inferred from the SEM imaging, see Fig. 1(a) of the main manuscript) and $\rho_{\mathrm{Pt}} \simeq 21450\ \mathrm{kg\ m^{-3}}$ the mass density of Platinum.

Supplementary Note 3

Dynamical backaction amplification

In the main text, we analyse the optomechanical dynamics in presence of a positive photothermal gradient ($k_p = \left(\frac{\partial F_p}{\partial x}\right)_{x_0} > 0$, with $x_0 = -\frac{w_0}{2}$), yielding to both a softening and cold damping of mechanical motion ($\delta\Omega_{\mathrm{p}} < 0$, $\Gamma_{\mathrm{p}} > 0$ and $T_{\mathrm{eff}} < T_0$, with $\delta\Omega_{\mathrm{p}}$ and Γ_{p} the optically induced frequency shift and damping rates, respectively, and with T_{eff} and T_0 the effective and ambient temperature). Following our linear dynamical backaction model, a reversed behaviour is expected when changing the sign of k_{p}. Due to the anti-symmetric nature of the photothermal force, such situation must be encountered on the other side of the optical waist, $k_{\mathrm{p}}\left(x_0 = \frac{w_0}{2}\right) < 0$. We subsequently move the hybrid CNT resonator to the average position $x_0 = \frac{w_0}{2}$ and further acquire the optomechanical fluctuation spectra for increasing input optical power. Supplementary Figure 1(a) shows three typical spectra, acquired for the input power being set to $70\ \mathrm{nW}$, $1.1\ \mu\mathrm{W}$ and $3\ \mu\mathrm{W}$, respectively. A clear amplification and narrowing is observed, as well as motion hardening ($\delta\Omega_{\mathrm{p}} > 0$). Supplementary Figure 1(b) and Supplementary Figure 1(c) show the analysis of Γ_{p} and $\delta\Omega_{\mathrm{p}}$ as inferred from fitting the optomechanical spectra. The linear, decreasing (resp. increasing) trends of Γ_{p} (resp. $\delta\Omega_{\mathrm{p}}$) as functions of the optical power are well confirmed. In particular, a slope $\left(\frac{\partial \Gamma_{\mathrm{p}}}{\partial P}\right)_{\frac{w_0}{2}} \simeq 7.3\ \mathrm{Hz}\ \mu\mathrm{W}^{-1}$, whose absolute value successfully compares to $\left(\frac{\partial \Gamma_{\mathrm{p}}}{\partial P}\right)_{-\frac{w_0}{2}} \simeq 6.2\ \mathrm{Hz}\ \mu\mathrm{W}^{-1}$ obtained on the cooling side. Last, the ratio of the slopes $\Gamma_{\mathrm{p}}/\delta\Omega_{\mathrm{p}}$ enables to determine $\tau \simeq 2.66 \times 10^{-6}s$, very close to the value obtained on the cooling side where a time constant $\tau \simeq 2.75 \times 10^{-6}s$ was found.
